# Metabolic breakdown: Linking insulin resistance and mitochondrial dysfunction to neurodegeneration in Alzheimer’s disease

**DOI:** 10.4103/NRR.NRR-D-25-00144

**Published:** 2025-06-19

**Authors:** Simona Lanzillotta, Lucrezia Romana Rolfi, Barbara Zulli, Eugenio Barone

**Affiliations:** Department of Biochemical Sciences “A. Rossi-Fanelli”, Faculty of Pharmacy and Medicine, Sapienza University of Rome, Rome, Italy

**Keywords:** aging, Alzheimer’s disease, brain insulin resistance, Down syndrome, energy metabolism, glucagon-like peptide 1, incretins, insulin, mitochondria, neurodegeneration

## Abstract

The increasing prevalence of metabolic disorders and neurodegenerative diseases has uncovered shared pathophysiological pathways, with insulin resistance and mitochondrial dysfunction emerging as critical contributors to cognitive decline. Insulin resistance impairs neuronal metabolism and synaptic function, fostering neurodegeneration as observed in Alzheimer’s disease and Down syndrome. Indeed, Down syndrome, characterized by the triplication of the *APP* gene, represents a valuable genetic model for studying early-onset Alzheimer’s disease and accelerated aging. Building on the link between metabolic dysfunctions and neurodegeneration, innovative strategies addressed brain insulin resistance as a key driver of cognitive decline. Intranasal insulin has shown promise in improving cognition in early Alzheimer’s disease and type 2 diabetes, supporting the concept that restoring insulin sensitivity can mitigate neurodegeneration. However, insulin-based therapies risk desensitizing insulin signaling, potentially worsening the disease. Incretins, particularly glucagon-like peptide 1 receptor agonists, offer neuroprotective benefits by enhancing insulin sensitivity, metabolism, and synaptic plasticity while reducing oxidative distress and neuroinflammation. This review focuses on current knowledge on the metabolic and molecular interactions between insulin resistance, mitochondrial dynamics (including their roles in energy metabolism), and oxidative distress regulation, as these are pivotal in both Alzheimer’s disease and Down syndrome. By addressing these interconnected mechanisms, innovative treatments may emerge for both metabolic and neurodegenerative disorders.

## Introduction

The growing prevalence of type 2 diabetes mellitus (T2DM) and Alzheimer’s disease (AD) has emerged as a pressing concern for public health, imposing significant burdens on societies worldwide. Long thought to be completely distinct pathologies, these conditions are now recognized as closely interconnected. Studies highlight shared pathophysiological mechanisms contributing to cognitive decline and metabolic dysfunctions, supporting the definition of AD as type-3 diabetes (Dierssen et al., 2020; Barone, 2022; Li et al., 2022). Chronic inflammation, oxidative stress, mitochondrial impairment, and vascular deterioration are important considerations in understanding how T2DM can promote neurodegenerative processes that contribute to the development of AD (Perluigi and Butterfield, 2012; Lanzillotta et al., 2021). Similarly, AD is marked by the gradual malfunctioning and death of specific groups of vulnerable neurons, frequently linked to the buildup of misfolded or clumped proteins, mitochondrial dysfunction, and oxidative distress (Tramutola et al., 2020).

At the heart of this relationship lies insulin, a hormone traditionally associated with glucose regulation that also exerts profound effects on brain health. Insulin resistance within the central nervous system has been increasingly identified as a major risk factor for neurodegenerative disorders. Alterations of insulin signaling can hinder neuronal metabolism, impair neuronal survival and plasticity, and compromise synaptic functions, thereby fostering cognitive deterioration (Arnold et al., 2018). This process is particularly concerning in the context of T2DM, where metabolic dysregulation is accompanied by a pronounced risk of cognitive decline and dementia, including AD (Holscher, 2019).

By deepening the biochemical interactions involved in the pathogenesis of these two pathologies, it becomes clear that the repercussions of insulin resistance go beyond metabolic dysfunction. In the brain, insulin facilitates essential processes such as the activation of neural growth regulators that support neuronal maintenance and neuroplasticity (Arnold et al., 2018). When insulin signaling is impaired, the ability of the brain to sustain these critical functions wanes, heightening the risk of neurodegeneration (Tramutola et al., 2020). Remarkably, emerging studies suggest that enhancing insulin sensitivity, potentially through pharmacological interventions, may hold promise for delaying cognitive decline in those with metabolic disorders (Ribaric, 2016; Pelle et al., 2023).

The BIR process becomes even more complex in individuals with Down syndrome (DS), the most common genetic condition compatible with life, marked by the presence of an extra copy of human chromosome 21 (Tramutola et al., 2020). DS not only predisposes individuals to intellectual disability but also represents a genetic form of AD due to the triplication of the *APP* gene located on chromosome 21 (Fortea et al., 2021).

The significant pathological overlap between DS and AD highlights DS as an invaluable model for exploring AD-associated neuropathological mechanisms. Individuals with DS experience an accelerated aging process, making them particularly suited for investigating the temporal progression of AD. Neuropathological changes associated with AD, such as amyloid deposition and tau pathology, emerge in DS brains decades earlier than in the general population, providing a unique opportunity to study the disease at its earliest stages. Furthermore, unlike sporadic AD, which results from a multifactorial interaction of genetic, environmental, and lifestyle factors, DS offers a genetically more uniform population (Fortea et al., 2021). This homogeneity reduces variability, enabling researchers to pinpoint specific pathways driving AD pathogenesis.

Additionally, the overexpression of several genes on chromosome 21, other than the *APP* gene, profoundly impacts metabolic processes and insulin regulation in DS. Alterations in glucose metabolism, such as insulin resistance or insufficient insulin production, disrupt insulin signaling pathways, thereby heightening the risk of neurodegenerative outcomes. A thorough understanding of these metabolic dysfunctions is crucial for developing targeted interventions that could enhance cognitive health and quality of life in individuals with DS (Fortea et al., 2021).

Overall, the intricate connection between metabolic disorders and neurodegenerative diseases highlights the need for a comprehensive examination of the mechanisms linking these conditions (Tran et al., 2024). As research advances in understanding the complex interplay between insulin resistance, cognitive decline, and AD, new therapeutic avenues are emerging that promise not only to address diabetes but also to safeguard and improve brain health in diverse populations.

This review aims to provide a comprehensive exploration of how metabolic dysfunctions, particularly insulin resistance and mitochondrial abnormalities, contribute to the pathophysiology of both sporadic and genetically predisposed forms of AD. Furthermore, we will cover the role of glucagon-like peptide 1 (GLP-1) and related signaling as alternative pathways under investigation in several clinical trials to improve brain functions and AD neuropathology. Remarkably, by integrating insights from DS as a genetic model for AD, we hope to uncover shared mechanisms and potential interventions that could mitigate the burden of neurodegenerative diseases.

## Search Strategy

In this narrative review, we searched articles in the PubMed database from October 2024 to January 2025, using the following keywords: insulin resistance, mitochondrial dysfunction, oxidative distress, Alzheimer’s disease, Down syndrome, brain insulin resistance, GLP-1 receptor agonists, mitochondrial quality control, and incretin signaling. Additional keywords included combinations of metabolism, neurodegeneration, and therapeutic interventions. Articles were screened based on titles and abstracts, and full texts were reviewed to ensure relevance to the core themes of this review. Only English-language publications were considered. We prioritized original research and review articles that addressed the mechanistic links between insulin signaling and mitochondrial dynamics in AD and DS. Several older references were included to contextualize fundamental discoveries. No restrictions were placed on study types.

## Complex Interplay Between Insulin Signaling and Mitochondrial Function in the Central Nervous System

### Insulin signaling

Insulin is a peptide hormone primarily produced by pancreatic β-cells, known for its roles in glucose and lipid metabolism, vascular regulation, and cell growth. Insulin also has a multifaceted role in the central nervous system (Neth and Craft, 2017).

The brain was considered for a long time an insulin-insensitive organ because glucose uptake in the brain is not primarily regulated by insulin (Hom and Goodner, 1984; Hasselbalch et al., 1999). However, growing evidence suggested that insulin can enhance glucose uptake in the spinal cord tissues and some brain regions, such as the prefrontal cortex, hippocampus, and hypothalamus (Havrankova et al., 1978; Ramnanan et al., 2013). Insulin readily crosses the blood–brain barrier via a receptor-mediated transport system, and the rate of transport can be modulated by various conditions, including obesity and inflammation (Rhea and Banks, 2019). In addition to glucose metabolism and energy balance in the brain, insulin is involved in other essential physiological processes, such as neural connectivity modulation, memory encoding, and cognition (Benedict et al., 2007; Taouis and Torres-Aleman, 2019). More recently, attention has focused on the involvement of insulin in brain aging, with concerns that disruptions in its regulatory functions may contribute to the onset of neurodegenerative diseases in later life (Craft and Watson, 2004).

**Figure 1:**
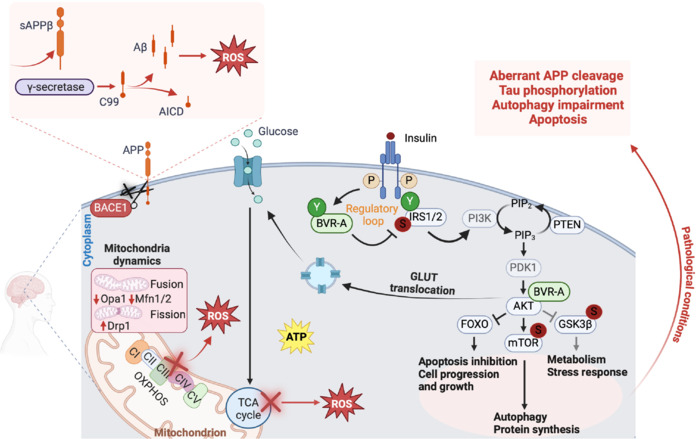
Role of insulin signaling in brain metabolic regulation: Physiological and pathological implications. Under physiological conditions, insulin binds to its receptor (IR), triggering autophosphorylation on tyrosine (Y) residues and activating receptor tyrosine kinase-mediated phosphorylation of its substrates, IRS1/2, at specific Y sites. In parallel, IR phosphorylates BVR-A, enabling it to function as a serine/threonine/tyrosine (S/T/Y). As part of a regulatory loop, BVR-A phosphorylates IRS1 on inhibitory S residues, preventing aberrant activation in response to insulin. This signaling cascade activates the PI3K-AKT pathway, which supports glucose uptake via GLUT translocation, cell survival and metabolism by inhibiting FOXO, protein synthesis and autophagy through mTOR activation, and glycogen metabolism and neuroprotection via GSK3β inhibition. Insulin also enhances glucose oxidation, fueling ATP production and maintaining MQC. Additionally, it regulates Aβ metabolism, protecting against AD and DS. Conversely, under pathological conditions such as BIR, this finely tuned signaling network is disrupted. IR signaling impairment leads to excessive IRS1 phosphorylation at inhibitory sites (S residues), preventing the PI3K-AKT activation and triggering detrimental downstream effects, including glucose uptake impairment, leading to reduced cellular energy availability; FOXO dysregulation, exacerbating oxidative distress and metabolic imbalance; mTOR hyper-activation, impairing autophagy and protein homeostasis; GSK3β over-activation, contributing to Tau hyperphosphorylation and neurodegeneration. Mitochondrial dysfunction further compounds these effects by reducing ATP synthesis, impairing MQC, and promoting structural and functional damage. This disruption in metabolic homeostasis weakens the brain’s ability to prevent Aβ accumulation, fostering neurodegenerative progression. Black arrows: activation of physiological processes involved in metabolism, mitochondrial function, and neuronal survival. Black lines: inhibition. Red arrows/lines: pathological alterations associated with BIR and neurodegenerative disease. Created with BioRender.com. AD: Alzheimer’s disease; AICD: amyloid precursor protein intracellular domain; AKT: protein kinase B; APP: amyloid precursor protein; ATP: adenosine triphosphate; Aβ: amyloid-beta; BACE1: beta-secretase 1; BVR-A: biliverdin reductase A; C99: C-terminal fragment of APP C99; CI–V: mitochondrial complexes I–V; Drp1: dynamin-related protein; DS: Down syndrome; FOXO: Forkhead box O; GLUT: glucose transporter; GSK3β: glycogen synthase kinase-3 beta; IR: insulin receptor; IRS1/2: insulin receptor substrate 1/2; Mfn1/2: mitofusin 1/2; MQC: mitochondrial quality control; mTOR: mechanistic target of rapamycin; Opa1: optic atrophy 1; P: phosphorylation; PDK1: pyruvate dehydrogenase kinase 1; PI3K: phosphatidyl inositol 3-kinase; PIP2: phosphatidylinositol 4,5-bisphosphate; PIP3: phosphatidylinositol (3,4,5)-trisphosphate; PTEN: phosphatase and tensin homolog; ROS: reactive oxygen species; S: serine residue; T: threonine residue; TCA cycle: tricarboxylic acid or krebs cycle; Y: tyrosine residue.

On a molecular level, insulin binds the insulin receptor (IR) and the insulin-like growth factor type 1 receptor, both of which are endowed with tyrosine kinase activity (Neth and Craft, 2017). The IR exists in two distinct isoforms: IR-A and IR-B. IR-A, present in the adult nervous system, has a higher affinity for insulin compared to IR-B, which is predominantly located in adipose tissue, liver, and skeletal muscle (Neth and Craft, 2017). The IR, a tyrosine kinase receptor, is located on the cell surface in α2β2 configuration. When insulin binds to the α-subunits, it activates the tyrosine kinase domain of the β-subunits, triggering a cascade of phosphorylation events crucial for initiating insulin signaling pathways that regulate various cellular processes (Andersen et al., 1995; Whittaker and Whittaker, 2005; **Figure 1**).

The mechanism of insulin signal transduction is modulated by the coordinated recruitment of several signaling proteins, which undergo phosphorylation events leading to their activation/inactivation, including the insulin receptor substrates (IRSs), the biliverdin reductase-A (BVR-A) protein (Cimini et al., 2022), the phosphatidylinositol 3-kinase (PI3K), the AKT protein (or protein kinase B) (Haeusler et al., 2018; Petersen and Shulman, 2018) and the Ras–mitogen-activated protein kinase (MAPK) family proteins (**Figure 1**). These proteins, conserved across insulin-responsive tissues, drive the insulin response. In the presence of insulin, most insulin responses are mediated by IRS-1 and IRS-2. IRS-1 regulates body growth and peripheral insulin action, while IRS-2 controls brain growth, body weight regulation, glucose homeostasis, and female fertility (White, 2003). IRS proteins are associated with the activation of two primary signaling pathways: the PI3K–AKT pathway, which mediates most of the metabolic effects of insulin, and the Ras–MAPK pathway, which is involved in the regulation of gene expression and works alongside the PI3K pathway to control cell growth and differentiation. Once IRS-1 is activated, it promotes the activation of PI3K, which catalyzes the generation of the lipid product phosphatidylinositol (3,4,5)-triphosphate. This leads to the activation of the kinase AKT by phosphoinositide-dependent kinase 1 (Czech, 1985; Taniguchi et al., 2006; Hubbard, 2013; Riehle and Abel, 2016; He et al., 2021; **Figure 1**).

AKT orchestrates a range of cellular functions through the phosphorylation of proteins that participate in: (i) inhibition of the forkhead transcription factor (Forkhead box O); (ii) inhibition of glycogen synthase kinase 3β (GSK-3β) and promotes glycogen synthesis; (iii) activation of mechanistic target of rapamycin (Zhang et al., 2011; Farhan et al., 2017; Manning and Toker, 2017; Glaviano et al., 2023; **Figure 1**). In this way, insulin stimulates the translocation of glucose transporter 4 from the intracellular compartment to the plasma membrane, enhancing glycolytic metabolism (Grillo et al., 2009; **Figure 1**). Stimulation of this pathway preserves mitochondrial membrane integrity and reduces the generation of free radicals that damage mitochondrial DNA and trigger pro-apoptosis mechanisms (Halestrap et al., 2000; **Figure 1**).

### Mitochondrion: Powerhouse and regulator of neuronal functions

Mitochondria are highly adaptable organelles that play a crucial role in sustaining cellular balance and stability. They mediate the cellular response in reaction to various stressors, including nutrient deficiency, oxidative distress, DNA damage, and endoplasmic reticulum stress (Spinelli and Haigis, 2018).

The main function of mitochondria is the production of adenosine triphosphate (ATP), hence their nickname “powerhouse of the cell.” The majority of ATP is generated by mitochondria through oxidative phosphorylation, a process that efficiently converts glucose into usable energy. In addition to mitochondrial ATP production, aerobic glycolysis in the cytoplasm provides a complementary pathway, particularly in regions with high metabolic demands or during specific physiological states (Casanova et al., 2023; **Figure 1**). Dysfunction in energy metabolism, particularly in mitochondrial oxidative phosphorylation (OXPHOS), has been increasingly linked to a range of neurodegenerative and metabolic disorders (**Figure 1**). Impaired mitochondrial function not only reduces ATP production but also generates excessive reactive oxygen species (ROS), leading to oxidative distress and neuronal damage (**Figure 1**). Conditions such as AD, Parkinson’s disease, and amyotrophic lateral sclerosis exhibit disrupted energy metabolism as a core feature (Cunnane et al., 2020). Similarly, systemic metabolic disorders such as T2DM exacerbate this vulnerability, as chronic hyperglycemia and insulin resistance impair glucose utilization in the brain, further aggravating neurodegenerative processes. When glucose availability is limited, the brain exhibits remarkably metabolic flexibility by utilizing alternative energy substrates. Ketone bodies, synthesized in the liver during periods of fasting or carbohydrate restriction, and lactate, produced by exercising skeletal muscle, serve as critical energy sources (Evans et al., 2017). These substrates are transported to the brain via the bloodstream and metabolized to support energy production, ensuring functionality under varying metabolic conditions (Evans et al., 2017). However, the role of mitochondria goes far beyond energy production. Mitochondria are also implicated in the production of ROS, redox molecules, and various metabolites; they regulate cell signaling, mediate cell death, and support biosynthetic pathways. These various functions make mitochondria key sensors of cellular stress, enabling cells to adapt effectively to environmental changes (Vyas et al., 2016). Cells maintain energy balance by metabolizing sugars, amino acids, and fatty acids, funneling them into the tricarboxylic acid cycle (**Figure 1**). This produces NADH and FADH2, which transport electrons to the electron transport chain in the inner mitochondrial membrane (**Figure 1**). As electrons move through the electron transport chain, protons are pumped into the intermembrane space, creating an electrochemical gradient. Protons then flow back through ATP synthase (complex V), producing ATP (Spinelli and Haigis, 2018). In low-oxygen conditions, cells shift to glycolysis for energy production (Wang et al., 2020).

Mitochondria have become a focus of intense research due to their vital roles in normal tissue function, disease mechanisms, and potential as targets for diagnosis and treatment. Central to this interest is the concept of mitochondrial quality control (MQC), which includes processes collectively known as biogenesis, dynamics, and mitophagy (Lanzillotta et al., 2025). The coordinated expression of nuclear and mitochondrial genes drives mitochondrial biogenesis, providing an adequate number of mitochondria to support energy needs of the cell (Liu et al., 2024; Perluigi et al., 2024). Mitochondrial dynamics, including fusion and fission, are essential for maintaining functional mitochondria. Mitochondrial fission serves different purposes depending on its nature. When fission occurs centrally, it increases the number of mitochondria, supporting cellular proliferation. Peripheral fission, conversely, helps remove damaged mitochondrial fragments, contributing to quality control (Kleele et al., 2021). In contrast, mitochondrial fusion facilitates the exchange of proteins, metabolites, and mitochondrial DNA (mtDNA), ensuring functional synchronization within the mitochondrial network. Fusion also enhances oxygen consumption, oxidative phosphorylation, and ATP production (Yao et al., 2019; Ng et al., 2021). Morphologically, fusion can create elongated, ring-shaped, or T-shaped mitochondria depending on the mode of fusion (Chan, 2020). Mitophagy, conversely, specifically eliminates damaged mitochondria, preventing the accumulation of defective organelles (Liu et al., 2024; Perluigi et al., 2024). Disruptions in any of these pathways exacerbate cellular dysfunctions (Liu et al., 2024; Perluigi et al., 2024), often leading to increased oxidative distress and metabolic disturbances, which further intensify the physiological alterations observed in neurodevelopmental and neurodegenerative disorders (Mancuso et al., 2011; Mollo et al., 2020; Liu et al., 2024; Perluigi et al., 2024).

Alterations of MQC have been strongly associated with various pathologies (Chen et al., 2023; Lanzillotta et al., 2025; **Figure 1**). In neurodegenerative diseases, for instance, the expression of fusion-related proteins such as optic atrophy 1 and mitofusin-1 and 2 (MFN1 and MFN2) is reduced, while fission-related proteins such as mitochondrial fission 1 protein and dynamin-related protein 1 are upregulated (Su et al., 2010). Moreover, amyloid-β (Aβ) has been shown to trigger nitric oxide production, leading to neuronal damage and excessive mitochondrial fission through S-nitrosylation of Drp1 (Cho et al., 2009). Similarly, in metabolic disorders such as obesity and T2DM, mitochondrial fragmentation, is a key early event, preceding the generation of ROS. This fragmentation is driven by Drp1 activation and Opa1 downregulation, further exacerbating mitochondrial dysfunction and contributing to disease progression (Lanzillotta et al., 2025; **Figure 1**).

Remarkably, the brain consumes up to 60% of the total energy available to the body during development (Steiner, 2019). This high energy requirement is due to the brain’s continuous need to maintain ion gradients across neuronal membranes, support synaptic transmission, and facilitate other cellular processes critical for cognition, memory, and overall brain function. While neurons rely on aerobic respiration for energy, astrocytes, which use about 5%–15% of the brain’s total energy, mainly utilize anaerobic glycolysis (Antunes et al., 2024). Hence, the perturbation of mitochondrial processes links directly to brain functions and the development of neurodegenerative diseases. It is worth mentioning that the brain is rich in polyunsaturated fatty acids, making it highly susceptible to oxidative and nitrosative damage that are associated with neurodegenerative diseases such as AD, where prolonged oxidative and nitrosative stress can exacerbate cell death and cognitive decline (Butterfield and Boyd-Kimball, 2020). The production rate of ROS is closely linked to mitochondrial membrane potential and the activity of electron transport chain complexes. Consequently, a dissipation of mitochondrial membrane potential, particularly under conditions where OXPHOS is impaired, can lead to increased ROS generation (Angelova and Abramov, 2018; Lushchak and Storey, 2021).

## Mitochondria and Insulin Signaling in Metabolic Disorders

Metabolic disorders are a heterogeneous group of conditions that affect metabolism, the process by which the body converts food into energy and substances needed for growth, repair, and cellular function. They can be hereditary or acquired and include dysfunctions of carbohydrate, lipid, protein, or nucleic acid metabolism. Among the most common are T2DM, obesity, and dyslipidemia.

Acquired metabolic disorders, such as T2DM and metabolic syndrome, are frequently associated with environmental factors and unhealthy lifestyles, including obesity, physical inactivity, and a diet rich in sugar and saturated fats. Insulin resistance is central to these conditions and is strongly associated with mitochondrial dysfunctions (Kim et al., 2008). These events trigger ROS production, as well as reduced fatty acids oxidation and the accumulation of free fatty acids in the cytosol, which, as in a vicious cycle, sustain the progression of these metabolic disorders (Amorim et al., 2022).

Skeletal muscle is the main target organ for insulin-stimulated glucose uptake, as well as glycogen production and storage, responsible for up to 70% of whole-body glucose disposal (DeFronzo and Tripathy, 2009). It also plays a central role in insulin resistance. In T2DM, insulin sensitivity in both skeletal muscle and the whole body is profoundly impaired, disrupting glucose metabolism regulation. This dysfunction contributes to abnormalities in glycolipid metabolism, including lipid synthesis, oxidation, and transport, along with glycogen synthesis and glucose transport. Additionally, it impairs mitochondrial function in skeletal muscle, leading to elevated blood glucose levels (Pinti et al., 2019).

Several lines of evidence show that muscle from obese subjects or T2DM patients are characterized by a tight link between insulin resistance and mitochondrial dysfunctions (Zorzano et al., 2009b). Within this frame, proteins that have been identified to participate in mitochondrial dynamism (the transport of mitochondria along the cytoskeleton and the control of their morphology and distribution, which are regulated by fusion and fission processes) also appear to be involved in metabolism. One of these proteins, MFN2, stimulates respiration and the oxidation of substrates. In this regard, the skeletal muscle tissue of individuals with obesity and T2DM exhibits reduced expression of MFN2 (Zorzano et al., 2009a). Consequently, disturbances in the activity of the proteins responsible for mitochondrial dynamics may contribute to the impairment of mitochondrial function observed in skeletal muscle of individuals with obesity and T2DM (Zorzano et al., 2009a). Zhang et al. (2015) investigated the role of miR-106b on palmitic acid (PA)-induced mitochondrial dysfunction and its association with insulin resistance in C2C12 myotubes. It is found that PA treatment increased miR-106b expression, while silencing miR-106b improved insulin sensitivity by increasing MFN2 levels, which are crucial for mitochondrial function. Additionally, the loss of miR-106b function partially restored mitochondrial morphology, enhanced mitochondrial DNA and ATP levels, and reduced ROS accumulation. The study also showed that silencing miR-106b upregulated the ERR-α/PGC-1α/MFN2 axis, which may help protect against PA-induced mitochondrial damage and insulin resistance (Zhang et al., 2015). Another group of researchers tested the hypothesis that high-fat diet (HFD) induced mtDNA damage, which in turn is linked to mitochondrial dysfunction, oxidative distress, and activation of endoplasmic reticulum stress-associated markers, protein degradation, and apoptosis in both skeletal muscle and liver in a mouse model (C57Bl/6j male mice) representing obesity-induced insulin resistance. Additionally, the high-fat diet significantly decreased the levels of two key proteins involved in insulin signaling, AKT and IRS-1 in skeletal muscle, and AKT in liver (Yuzefovych et al., 2013).

Remarkably, these alterations might be reverted by high-intensity interval training that promotes significant effects on glycolipid metabolism and mitochondrial dynamics in the skeletal muscle of mice in which insulin resistance was induced by a HFD or by streptozocin (Zheng et al., 2020). The results showed that high-intensity interval training significantly reduced body weight, fat mass, fasting blood glucose, and serum insulin in diabetic mice. It also improved glucose tolerance and insulin tolerance. Additionally, mitochondrial morphology and quantity improved, and proteins related to mitochondrial biosynthesis and dynamics were upregulated (Zheng et al., 2020).

Other than skeletal muscle, the liver plays a vital role in maintaining whole-body glucose balance. Insulin resistance is a characteristic trait of non-alcoholic fatty liver disease (NAFLD). Consistent with their central role in fatty acid metabolism and energy homeostasis, mitochondrial dysfunction is implicated in the pathogenesis of NAFLD and insulin resistance (Garcia-Ruiz et al., 2013).

Several studies also focus on analyzing mitochondrial function in conditions of fatty liver and insulin resistance by examining mitochondria isolated from the liver of obese rats or HFD-induced, to better understand the mechanisms involved in these metabolic alterations. Indeed, it has been hypothesized that insulin resistance represents the “first strike” in the development of hepatic steatosis, with steatosis subsequently aggravated by inflammation and oxidative distress (Day and James, 1998). A key study demonstrates that mitochondrial dysfunction in the liver occurs before the onset of NAFLD and insulin resistance in the Otsuka Long-Evans Tokushima Fatty (OLEFT) rats (Rector et al., 2010). This suggests that mitochondrial dysfunction may be either a cause or a consequence of the development of NAFLD.

An interesting work linking metabolic changes in the liver to brain alterations was conducted by Wang et al. (2019), who evaluated the effects of lycopene - a carotenoid with antioxidant and anti-obesity properties (Palozza et al., 2008; Zhu et al., 2020) - on mitochondrial metabolism, synaptic dysfunction, and inflammation. Collectively, these results showed that supplementation mitigated LPS-induced neuronal damage and synaptic dysfunction. Lycopene increased the expression of neurotrophic factors and synaptic proteins and improved insulin resistance and mitochondrial dysfunction in both the brain and liver. Additionally, lycopene reduced neuroinflammation, hepatic inflammation, and circulating levels of inflammatory cytokines and insulin (Wang et al., 2019).

## Mitochondria and Insulin Signaling in Alzheimer’s Disease

AD is a complex progressive neurodegenerative condition that affects millions of people worldwide (No authors listed, 2023), characterized clinically by a gradual decline in cognitive function, memory loss, behavioral and personality changes, and pathologically by the presence of extracellular Aβ plaques, intracellular deposition of neurofibrillary tangles, Tau protein degeneration and significant neuronal loss (DeTure and Dickson, 2019; Breijyeh and Karaman, 2020). A growing attention on the role of metabolism, particularly the relationship between insulin, mitochondria, and the neurodegeneration observed in AD patients raised in recent years (Correia et al., 2012; Wang et al., 2017). The discovery that AD might be considered a form of “type 3 diabetes” has opened new perspectives on understanding the role of insulin and mitochondrial dysfunction in the disease (de la Monte and Wands, 2008; Nguyen et al., 2020b; **Figure 1**).

Insulin plays a crucial role in modulating brain functions such as food intake, energy expenditure, and cognition by interacting with insulin and insulin-like growth factor type receptors (Plum et al., 2005; Kleinridders, 2016). In addition, in AD a condition known as BIR is observed, and it describes the reduced ability of neurons to respond to insulin (Kellar and Craft, 2020; Nguyen et al., 2020a). This impairs synaptic function, increases neuro-inflammation, and reduces the brain’s ability to use glucose, leading to cognitive deficits (Spinelli et al., 2019; Liu et al., 2022a). Diminished insulin activity is also linked to increased production and accumulation of all hallmarks of AD; taken together, these processes are critical to the development and progression of the disease (Schell et al., 2021).

A relevant study (Talbot et al., 2012), conducted on the brains of patients with mild cognitive impairment (MCI) and AD, highlights BIR as a critical factor in AD, even in the absence of diabetes. The research reveals a significant impairment in insulin signaling at the IRS-1 level, with progressively elevated phosphorylation of IRS-1 at serine, which is observed in individuals with normal cognition, MCI, and AD, regardless of diabetes or APOE ε4 status. These insulin resistance biomarkers are found to correlate with the presence of oligomer Aβ plaques and negatively influence memory function. The findings suggest that BIR is an early and widespread feature of AD, driving cognitive decline independently of the traditional pathological hallmarks of the disease.

In another study (Bartl et al., 2013), an immunohistochemical evaluation was used to examine post-mortem brains (prefrontal cortex and hippocampus) from AD patients, individuals with AD associated with T2DM, those with T2DM alone, and age-matched controls. The researchers assessed the number of cells expressing the insulin receptor β-subunit and phosphorylated peroxisome proliferator activated receptors (PPARγ(p)) (nuclear receptors, one of the classes of drugs used to treat insulin resistance, appear to have anti-inflammatory effects (Combs et al., 2000)). The results revealed that AD patients had significantly fewer insulin receptor β-subunit-positive cells compared to all other groups across all brain regions. In contrast, PPARγ(p)-positive cells were significantly more abundant in all patient groups compared to the controls. Although T2DM and AD are not directly linked, they share common histological features, such as reduced insulin receptor β-subunit-positive cells and increased PPARγ(p)-positive cells in all brain regions. These findings may help explain the higher prevalence of AD observed in elderly diabetic patients.

Arvanitakis et al. (2020) conducted a similar study on individuals (male and female) with and without diabetes. They used enzyme-linked immunosorbent assay, immunohistochemistry, and *ex vivo* stimulation of brain tissue with insulin, and they assessed insulin signaling in the post-mortem frontal cortex, whose data documented AD neuropathology. In particular, they assessed key proteins involved in the signaling, such as IRS-1 and AKT. Results showed that IRS-1 serine phosphorylation and serine/threonine-AKT phosphorylation levels were similar in both diabetic and non-diabetic patients. Phosphorylation of AKT was found to be correlated with the overall neuropathology score of AD, whereas phosphorylation of IRS-1 showed no association with AD.

Significant mitochondrial alterations play a central role in neuronal degeneration. In AD, this dysfunction manifests as reduced energy production, increased ROS levels, and excessive fragmentation (Misrani et al., 2021). When insulin signaling is impaired in the brain, neurons have a reduced capacity to handle oxidative distress and regulate energy metabolism, leading to further mitochondrial dysfunction, which in turn exacerbates insulin resistance (Galizzi and Di Carlo, 2022). Several studies have shown that mitochondrial dysfunction can interfere with insulin receptor phosphorylation and its downstream signaling pathways, reducing the efficacy of insulin signaling. Recently, Lanzillotta et al. (2024b) have established that reduced BVR-A protein levels disrupt insulin signaling and mitochondrial bioenergetics in the brain (for review see Cimini et al., 2022). Loss of BVR-A causes the hyper-activation of IRS-1 but impairs the AKT-GSK-3β pathway, promoting the uncoupling among proteins belonging to the insulin signaling, and preventing the accumulation of pGSK-3β^S9^ in mitochondria. This event worsens mitochondrial metabolism by inhibiting oxidative phosphorylation while triggering the mitochondrial unfolded protein response and AD-related pathology (Triani et al., 2018; Barone et al., 2019; Sharma et al., 2019; Lanzillotta et al., 2024b).

Additionally, dysfunctional mitochondria contribute to neuro-inflammation, another factor that impairs insulin sensitivity in the brain (van Horssen et al., 2019). This detrimental feedback loop between insulin and mitochondria not only leads to neurodegeneration but also promotes the accumulation of Aβ and Tau, accelerating AD progression. To prove that mitochondria represent a functional link between insulin resistance and AD, studies by Carvalho et al. (2012) demonstrated that mitochondria from the brains of 3xTg-AD and sucrose-treated wild-type mice exhibited comparable disruptions in the respiratory chain and phosphorylation system, reduced ability to accumulate calcium, ultrastructural defects, and increased oxidative stress. Sucrose-treated wild type mice also had elevated Aβ protein levels, a key marker of AD (Yao et al., 2009; Carvalho et al., 2012). These findings suggest that diabetes-related metabolic changes contribute to AD-like pathology in mice.

Gabrielli et al. (2024) highlighted the importance of mitochondrial dysfunction in AD by addressing the question on whether mitochondrial dysfunction might represent a disease cause or consequence. They treated SH-SY5Y cells and induced pluripotent stem cell-derived neurons with chloramphenicol, an antibiotic that inhibits mtDNA-generated transcript translation. The results showed that cells responded by increasing mtDNA copy number and transcription. Nuclear-expressed respiratory chain mRNA and protein levels also changed. There were AD-consistent concordant and model-specific changes in amyloid precursor protein, Aβ, apolipoprotein E, Tau, and α-synuclein biology. Primary mitochondrial dysfunction induced compensatory organelle responses, changes nuclear gene expression, and alters the biology of AD-associated genes and proteins in ways that may recapitulate brain aging and AD molecular phenomena. Hence, mitochondrial stress creates an environment that promotes AD pathology (Gabrielli et al., 2024).

These results appear of interest, particularly considering the role insulin signaling in mitochondrial functions, suggesting that insulin resistance may also create a favorable environment for the development of AD pathology.

Another critical aspect of the interaction between insulin and mitochondria is oxidative distress (Perluigi et al., 2024). Oxidative distress negatively affects the ability of the brain to regulate neuroplasticity and synaptogenesis, two processes vital for learning and memory. This is particularly relevant because neurons, unlike other cells, are highly sensitive to oxidative damage, with limited regenerative capacity and antioxidant defenses (Tonnies and Trushina, 2017).

Research from our laboratory (Di Domenico et al., 2017) and others (Butterfield and Lange, 2009; Butterfield and Halliwell, 2019) has highlighted the strong connection between disruptions in redox balance and AD pathology, revealing redox-regulated events that may contribute to both AD onset and progression (Di Domenico et al., 2017). Given the complexity of AD mechanisms, further understanding of intracellular pathways affecting redox homeostasis is essential. This knowledge could guide the development of treatments targeting oxidative distress-related toxicity, potentially slowing AD progression and enhancing the quality of life for those affected.

AD is considered one of the main causes of disability in the elderly population: aging is the most significant risk factor for sporadic or late-onset AD. Typically emerge after the age of 65. Alongside aging, one of the determinant key factors for the development of late-onset AD is the presence of the *ApoE* ε4 allele, a variant of the apolipoprotein E (*APOE*) gene (Kanekiyo et al., 2014; Guo et al., 2020). The relationship between metabolic disorders, the APOE ε4 allele, and AD is a growing area of research, but the mechanisms by which these factors interact remain complex and not fully understood. Notwithstanding, studies have indicated that individuals carrying this allele have a higher likelihood of developing Alzheimer’s compared to non-carriers (Liu et al., 2013). However, recent evidence suggests that the presence of T2DM can further exacerbate this risk, particularly in individuals with the APOE ε4 allele (Raulin et al., 2022).

Late-onset AD affects about 95% of patients (Association, 2019), while familial AD (FAD), also known as early-onset AD or autosomal dominant AD, represents less than 5% (Association, 2019). FAD is caused by mutations in three specific autosomal genes directly responsible for Aβ production: amyloid precursor protein (APP), presenilin1 (PSEN1 or PS1), and presenilin2 (PSEN2 or PS2) (Association, 2019). Since FAD is a rare condition, studying the brains of affected individuals during preclinical and early clinical stages is a challenge. Currently, the sequential analysis of specific parameters, such as APP, PS1, and PS2 in transgenic mice represents an invaluable tool for uncovering mechanistic insights into disease progression in FAD (Aso et al., 2012; Chen et al., 2013; Szaruga et al., 2015; Liu et al., 2022b).

There is a notable gap in the literature concerning the link between mitochondrial alterations and FAD. A recent review (Wang and Zhang, 2021) sheds light on this topic, discussing the role of mitochondria-associated endoplasmic reticulum membranes in AD pathogenesis. Specifically, it highlights how mitochondria-associated endoplasmic reticulum membranes - key microdomains at the interface of the endoplasmic reticulum and mitochondria - regulate lipid metabolism, calcium homeostasis, and autophagy. Disruptions in these processes, common in AD, underline the potential significance of mitochondria-associated endoplasmic reticulum membranes in both FAD and sporadic AD (Wang and Zhang, 2021).

## Mitochondria and Insulin Signaling in Down Syndrome

DS is the most common type of trisomy, congenital anomaly, and genetic cause of intellectual disability. Individuals with DS exhibit a distinct set of recognizable facial characteristics, along with various medical conditions such as congenital heart defects, gastrointestinal issues, hematological disorders, and immune system dysregulation, all resulting from an extra copy of chromosome 21 (Antonarakis et al., 2020). In addition, to various morphological and physiological characteristics, trisomy of human chromosome 21 is linked to cognitive impairment (Lott and Dierssen, 2010). Furthermore, most individuals with DS (after the age of 40 years) experience premature brain aging, early-onset cognitive decline, and an increased risk of developing AD (Dierssen et al., 2020), driven by the triplication of several genes, i.e., APP, and DYRK1A, on chromosome 21 (Fortea et al., 2021). This aspect fosters age-associated deficits of the proteolytic system that may further exacerbate the accumulation of oxidized/misfolded/polyubiquitinated proteins, including Aβ-peptide oligomers into senile plaques, as well as hyper-phosphorylated Tau into neurofibrillary tangles (Tramutola et al., 2017; Kennedy et al., 2025).

Therefore, DS is now considered a genetically determined form of AD. Additionally, DS is associated with higher rates of metabolic disorders, such as obesity, glucose intolerance, and T2DM, which further exacerbate brain dysfunctions (Barone et al., 2017, 2018; Dierssen et al., 2020).

As observed in the above sections, dysregulation of brain insulin signaling, characterized by alterations in cellular processes that regulate survival and neuronal plasticity, is a fundamental abnormality in AD. BIR greatly contributes to AD development in the general population. Research conducted by our team and others showed an early accumulation of insulin resistance markers in the DS brain, detectable in the brain during childhood, often preceding the onset of AD symptoms (Rivera et al., 2005; Tramutola et al., 2015, 2020). In individuals with DS, this can lead to problems like impaired glucose tolerance and increased risk of developing T2DM. Insulin resistance and mitochondrial dysfunction are interconnected in DS: insulin helps to regulate mitochondrial function, but when cells are resistant to insulin, mitochondrial activity can be impaired (Kim et al., 2008).

Previous studies have demonstrated how the levels and the activation of proteins involved in insulin signaling in the frontal cortex of Ts65dn (Lanzillotta et al., 2021), in the hippocampus of Ts66Yah (Lanzillotta et al., 2024a) mice (a model for DS) and euploid mice vary across different ages. Their findings revealed that Ts65dn and Ts66Yah mice exhibited a general dysfunction in (i) proteins regulating brain energy metabolism, (ii) markers of oxidative distress, and (iii) proteins involved in synaptic plasticity mechanisms.

Tramutola et al. (2023), on the other hand, tested the effects promoted by intranasal administration of KYCCSRK peptide in Ts2Cje mice (another DS mouse model), known to promote activation of insulin signaling. The study found that the KYCCSRK peptide successfully restored insulin signaling, increased mitochondrial complex levels, and reduced oxidative distress in the brains of Ts2Cje mice.

Mitochondrial dysfunction and oxidative distress are common features of DS, but the exact mechanisms behind these abnormalities are unclear. Another study performed by Helguera et al. (2013) explored the connection between altered energy metabolism and oxidative distress with changes in the transcription and function of primary human cortical neuronal and astrocyte cultures from DS control brain samples. Impaired mitochondrial activity is linked to changes in mitochondrial shape, and although enhancing mitochondrial fusion corrected the morphology, it did not resolve the functional issues.

In this context, Lanzillotta et al. (2025) investigated the mitochondrial unfolded protein response and MQC mechanisms in DS, focusing on their implications in redox homeostasis in the frontal cortex isolated from Ts2Cje mice, across different developmental stages. This study revealed that impaired mitochondrial unfolded protein response correlates with decreased mitochondrial activity, as evidenced by reduced oxygen consumption rates and altered OXPHOS complexes expression, alongside increased oxidative distress. Moreover, early-stage defects in MQC, including impaired biogenesis, accelerated mitochondrial fragmentation, and the activation of mitophagy were observed. Mitochondrial deficits in DS result mainly from an impaired ability to generate ATP through OXPHOS, along with decreased respiratory capacity, impaired membrane potential, and disturbed mitochondrial dynamics (Dierssen et al., 2020).

Recent studies by our research group (Perluigi et al., 2022) and others (Eren et al., 2020; Blommer et al., 2023; Malaguarnera and Cabrera-Pastor, 2024) have highlighted that the analysis of fluid biomarkers for cognitive impairment offers the advantage of being relatively non-invasive and able to monitor the health of neurons and other brain cells in real time. In particular, Perluigi et al. (2022) showed that BIR develops early in individuals with DS, independent of peripheral alterations, based on the analysis of neuronal‐derived extracellular vesicles, isolated from healthy donors and DS children from 2 to 17 years of age. The disruption of insulin signaling and the mechanistic target of rapamycin pathway represent an early event in the DS brain and likely contributes to cell energy dysmetabolism, mitochondrial dysfunction, and intellectual disability commonly observed in this population. These findings suggest that impaired insulin signaling could play a significant role in the neurological deficits associated with DS.

## Glucagon Like Peptide-1 Signaling in Metabolic Disorder, Mitochondrial Function Linked to Neurodegenerative Diseases

**Figure 2:**
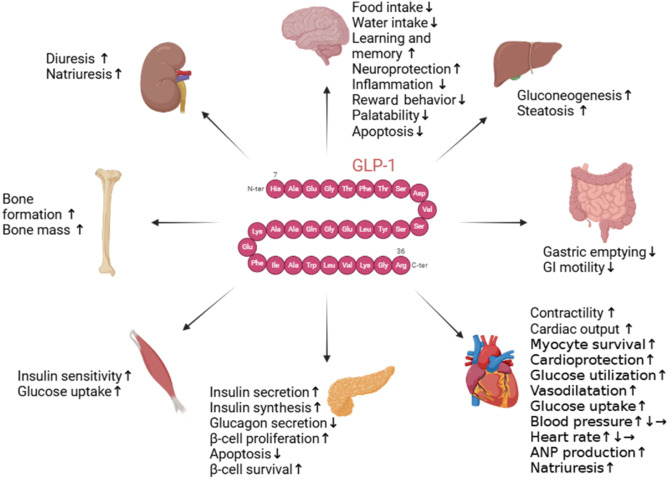
Schematic depiction of major targets of GLP-1 action. The image illustrates the multifaceted role of GLP-1 on its primary target tissues and organs. This hormone acts on glucose homeostasis, insulin secretion, inhibition of glucagon release, modulation of gastric emptying in the gastrointestinal tract, appetite regulation in the central nervous system, and shows potential cardioprotective and renal benefits. Created with BioRender.com. GLP-1: Glucagon-like peptide 1.

The link between insulin signaling, as previously described, and GLP-1 is fundamental in regulating glucose homeostasis and the function of pancreatic β-cells. GLP-1 is an incretin hormone constituted 30-amino acid or 31-amino acid and it is secreted by specialized enteroendocrine cells, in the distal intestine (L cells), in the presence of nutrients. In response to food intake, GLP-1 stimulates insulin secretion in a glucose-dependent manner, slows gastric emptying, inhibits food intake, increases natriuresis and diuresis, and modulates β-cell proliferation in rodents. Additionally, GLP-1 exhibits cardio- and neuroprotective effects, reduces inflammation and apoptosis, and influences processes such as learning, memory, reward behavior, and palatability (**Figure 2**). GLP-1, by acting via its receptors (GLP-1Rs) placed on pancreatic islet β-cells, is responsible for the incretin effect, which amplifies insulin secretion in response to ingested glucose (Ansari et al., 2024). A study highlighted that GLP-1 enhances the transcription and biosynthesis of the insulin gene through the activation of both protein kinase A (PKA)-dependent and -independent signaling pathways (described in details below) (Diz-Chaves et al., 2024). Specifically, GLP-1 stabilizes preproinsulin mRNA by increasing insulin expression (Portha et al., 2011).

Gupta et al. (2023) and others (Ansari et al., 2024) have shown the distribution, function, and potential implications of GLP-1 and its receptor in the human brain. As previously reported, GLP-1 and GLP-1R are expressed in various brain regions, including areas associated with memory, learning, and emotional regulation, such as the hippocampus, cortex, and amygdala. In this study, they saw that GLP-1 expression was detected across most cortical areas, with the highest levels in the frontal, prefrontal, and parietal cortices, as well as in the diencephalon and brainstem. The cerebellum, however, showed no GLP-1 and GLP-1R expression. GLP-1R expression peaked in the frontal cortex, while the orbitofrontal cortex and cerebellum showed minimal expression. Although the hippocampus displayed notable GLP-1R presence, GLP-1 immunoreactivity was inconsistent. Additionally, GLP-1 levels exhibited an age-related decline, with cortical expression nearly undetectable by the age of 60, except in the prefrontal cortex, while remaining detectable in subcortical regions (Gupta et al., 2023).

Llewellyn-Smith et al. (2011) shed light how medullary neurons, which mainly innervate brain regions involved in autonomic control, are capable of synthesizing GLP-1. Thus, central preproglucagon neurons are uniquely positioned to modulate sympathetic and parasympathetic outflow through inputs at a variety of autonomic locations within the central nervous system.

A study conducted by Alvarez et al. (2005) has demonstrated that the *GLP-1R* gene is expressed in the human brain as a functional protein with binding properties similar to those found in peripheral tissues. Moreover, the findings suggest that GLP-1 (7-36) amide (a biologically active form of GLP-1) interacts with this receptor to regulate glucose metabolism in specific brain regions associated with glucose sensing, such as the hypothalamus and brainstem. It has been observed that these areas contain neurons that respond to changes in blood glucose levels (Alvarez et al., 2005). Additionally, as previously mentioned (Gupta et al., 2023; Ansari et al., 2024), the expression of GLP-1R in these regions supports the direct role of GLP-1 in modulating glucose-dependent functions.

**Figure 3:**
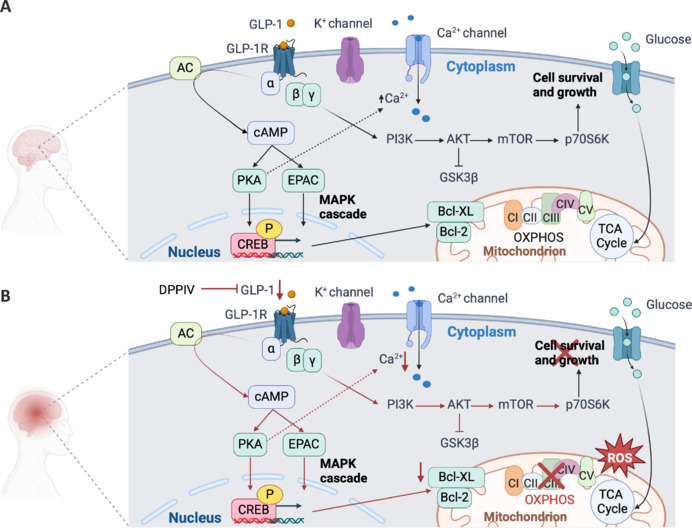
GLP-1 signaling pathway in the brain. (A) Physiological conditions. The binding of GLP-1 to its receptor (GLP-1R) initiates a signaling cascade that regulates multiple intracellular pathways, promoting neuronal survival and functions. Activation of the AC-cAMP-PKA pathway leads to phosphorylation of CREB, a transcription factor essential for neuroplasticity and cell survival. CREB activation induces the transcription of anti-apoptotic factors (Bcl-2, Bcl-XL), safeguarding neurons against cell death. In parallel, increased Ca²⁺ influx enhances neurotransmitter release and synaptic plasticity mechanisms. Additionally, EPAC activation stimulates the MAPK cascade, supporting long-term gene regulation, cellular repair, and neuroprotection. The PI3K-AKT pathway, activated via Gβγ subunits, converges with insulin signaling, reinforcing neurotrophic and metabolic homeostasis. (B) Pathological conditions. GLP-1 signaling impairment disrupts these protective mechanisms. AC-cAMP-PKA pathway inactivation prevents CREB phosphorylation, reducing transcription of anti-apoptotic genes and promoting neuronal apoptosis and neurodegeneration. Additionally, decreased Ca²⁺ influx leads to impaired neurotransmitter release and weakened synaptic plasticity. EPAC inactivation halts MAPK cascade signaling, reducing cellular repair and increasing vulnerability to neurodegenerative stressors. Furthermore, PI3K–AKT pathway inhibition exacerbates BIR, impairing neuronal metabolism and survival. Black arrows: physiological activation of neuroprotective pathways. Red arrows: pathological alterations contributing to neurodegeneration. Created with BioRender.com. AC: Adenylate cyclase; ADP: adenosine diphosphate; AKT: protein kinase B; ATP: adenosine triphosphate; Bcl-2: B-cell lymphoma 2; Bcl-XL: B-cell lymphoma-extra large; Ca²⁺: calcium ion; cAMP: cyclic adenosine monophosphate; CI–V: mitochondrial complexes I–V; CREB: cAMP response element-binding protein; CV: complex V; DPPIV: dipeptidyl peptidase IV; EPAC: exchange protein directly activated by cAMP; GLP-1: glucagon-like peptide 1; GLP-1R: glucagon-like peptide 1 receptor; GSK3β: glycogen synthase kinase 3 beta; K⁺: potassium ion; MAPK: mitogen-activated protein kinase; mTOR: mammalian target of rapamycin; OXPHOS: oxidative phosphorylation; P: phosphorylation; p70S6K: ribosomal protein S6 kinase beta-1; PI3K: phosphoinositide 3-kinase; PKA: protein kinase A; ROS: reactive oxygen species; TCA: tricarboxylic acid or Krebs.

As previously demonstrated by Alvarez et al. (2005) and Ansari et al. (2024), the binding between GLP-1 and GLP-1Rs activates an enzymatic cascade in the brain (**Figure 3A**). The activation of the GLP-1R, coupled to the Gα subunit, triggers the adenylate cyclase signaling cascade. This process catalyzes the conversion of ATP into cyclic adenosine monophosphate (cAMP), a key second messenger in cellular signaling. The resulting increase in cAMP levels leads to the activation of PKA, a critical enzyme that mediates numerous downstream effects. One of primary actions of PKA is the phosphorylation of the cAMP-response element-binding protein (CREB), a transcription factor involved in the regulation of genes essential for neuronal plasticity and survival. In parallel, adenosine diphosphate, a metabolic byproduct of adenylate cyclase activity, exerts its influence on potassium (K^+^) channels. This interaction promotes the depolarization of the neuronal membrane by closing K^+^ channels and reducing the rate of repolarization. As a result, voltage-dependent L-type calcium (Ca^2+^) channels are activated, allowing an influx of Ca^2+^ into the cell (**Figure 3A**). The rise in intracellular Ca^2+^ concentration plays a pivotal role in facilitating the release of neurotransmitters into the synaptic cleft, thereby modulating synaptic communication and plasticity. An alternative cAMP-mediated signaling pathway involves the activation of cAMP-regulated guanine nucleotide exchange factors, commonly referred to as EPAC (exchange protein directly activated by cAMP). Epac activation stimulates the MAPK cascade, initiating a slower but sustained transcriptional response (**Figure 3A**). This mechanism contributes to the long-term regulation of genes associated with cellular repair, growth, and differentiation. Furthermore, the Gβγ subunits associated with GLP-1R can independently activate PI3K. The PI3K/AKT signaling pathway represents an additional route activated by GLP-1R signaling. AKT, a central kinase in this pathway, phosphorylates multiple downstream substrates to regulate diverse cellular functions. These include the promotion of autophagy, enhancement of synapse formation, and facilitation of long-term potentiation, a mechanism critical for learning and memory (**Figure 3A**). Simultaneously, AKT exerts neuroprotective effects by inhibiting proinflammatory cytokine secretion, suppressing apoptotic pathways, and mitigating pathological processes such as Tau hyperphosphorylation and the accumulation of neurotoxic proteins such as α-synuclein and Aβ.

Through these multifaceted pathways, GLP-1R signaling not only supports synaptic activity and plasticity but also contributes to the broader maintenance of neuronal health and resilience in the face of injury or disease (Diz-Chaves et al., 2024).

The dipeptidyl peptidase IV (DPPIV) is a serine protease that specifically cleaves short peptides, whose penultimate amino acid in the NH_2_-terminal sequence is occupied by (hydroxy) proline or an alanine residue. Cleavage of the NH_2_-terminal region by DPPIV generates biologically inactive GLP-1, to prevent it from binding to GLP-1R to exert its physiological action (Metzemaekers et al., 2016; **Figure 3B**).

Inhibitors of this enzyme are known as glyptins (i.e., sitagliptin, alogliptin, linagliptin, and saxagliptin); they have been developed as antidiabetic drugs used for the treatment of T2DM due to their ability to prolong the half-life of GLP-1, thus achieving an overall prolonged and more intense hypoglycemic effect. Although DPPIV inhibitors do not cross the blood–brain barrier under normal conditions, this pharmacokinetic feature might limit their use in treating neurodegenerative diseases (Du et al., 2022). However, GLP-1 analogs with longer half-lives can penetrate the brain more effectively, providing therapeutic potential for neurodegenerative diseases due to their neuroprotective properties and ability to regulate feeding behavior (Rowlands et al., 2018).

Research highlights that GLP-1 plays a crucial role in protecting neurons, reducing oxidative distress, and supporting energy homeostasis, primarily through its influence on mitochondrial activity. These actions are critical because mitochondrial dysfunction is a hallmark of neurodegenerative diseases, contributing to cognitive decline, synaptic loss, and neuroinflammation (Grieco et al., 2019; **Figure 3B**).

The role of GLP-1 in mitochondrial function is being studied extensively, as it may have implications for neurodegenerative diseases (Monti et al., 2022b). However, only relatively few lines of research have addressed this topic, focusing on the activity of GLP-1 receptor agonists (GLP-1RAs), which improve mitochondrial dynamics, integrity, and functionality and that are critically addressed in the subsequent section. Overall, these improvements include enhanced fatty acid oxidation and reduced oxidative distress.

GLP-1 has been found to promote mitochondrial biogenesis and maintain mitochondrial membrane potential, playing a crucial role in cellular energy regulation and neuroprotection, particularly in astrocytes and neurons (Sposito et al., 2018). Indeed, GLP-1RAs have been shown to enhance insulin signaling and protect neurons from apoptosis, thereby improving cognitive functions (Rowlands et al., 2018).

Siddeeque et al. (2024) have highlighted in their study that neuroprotective effects of GLP-1RAs can be observed in obese patients. More specifically, this research has shown that GLP-1-based anti-diabetic drugs not only regulate blood glucose but also offer diverse benefits, including protecting pancreatic β-cells from apoptosis in T2DM. This study further explores the effects of these drugs, revealing a notably lower risk of developing AD among GLP-1RA users. The neuroprotective effects of GLP-1RAs are believed to work through multiple pathways that jointly protect neural tissue. These compounds modulate microglial activation and reduce the levels of pro-inflammatory cytokine, fostering an anti-inflammatory state. They also enhance mitochondrial function by increasing mitochondrial respiration and ATP synthesis, which is essential for high-energy demanding neural tissues. Activation of the nuclear factor erythroid 2-related factor 2 pathway and upregulation of antioxidant enzymes further shield against oxidative distress, while mitigating the aggregation of dysfunctional proteins is a key feature of neurodegeneration. Preclinical evidences suggest improvements in synaptic density, activity, motor function, dopaminergic neuron health, cortical function, and brain energy consumption. The positive effects on metabolic health and vascular function associated with GLP-1RA use may also support brain health and resilience against neurodegenerative processes (Siddeeque et al., 2024).

GLP-1R agonist also exhibit neuroprotective properties by downregulating the formation of Aβ plaques and tau protein phosphorylation in AD (Monti et al., 2022a).

A recent study conducted by Barrett et al. (2024) has shown that the reduced availability of GLP-1R in the caudate nucleus may be associated with AD. GLP-1R normally aids in metabolic regulation and neuroprotection, but its decrease in AD patients could harm neuronal function and contribute to cognitive decline. Furthermore, previous research has shown that the activation of GLP-1R can improve cognitive and neuroprotective functions, so its reduced presence in the caudate nucleus might exacerbate AD symptoms.

Xie et al. (2021) demonstrated that GLP-1, through its analog liraglutide, exerts neuroprotective effects in AD by improving astrocytic mitochondrial function. The mechanism involves activating the cAMP/PKA pathway, which prevents mitochondrial dysfunction, reduces oxidative distress, and enhances energy production in astrocytes exposed to Aβ. As a result, GLP-1 mitigates neuronal loss and supports cell viability, suggesting a potential therapeutic role in AD.

Vogrinc et al. (2024) reported that genetic variability in GLP-1R affects the onset of AD and is also associated with cerebrospinal fluid biomarkers of AD in patients with AD. The GLP-1R polymorphism rs10305420 is associated with the development of AD. Furthermore, both GLP-1R polymorphisms (GLP-1R rs10305420, GLP-1R rs6923761) investigated are associated with the levels of AD biomarkers in cerebrospinal fluid, thus supporting their role in AD pathology.

In the context of DS, where accelerated aging and BIR are prevalent, GLP-1 could modulate mitochondrial function, offering a new avenue for therapeutic intervention to slow down disease progression. Day et al. (2019) reported that with systemic administration of GLP-1 (specifically 9-36 fragment) in Ts65Dn (a DS mouse model), both females and males showed a decreased mitochondrial oxidative distress in the hippocampus, an improved dendritic spine structure and integrity, as well as an increase in mature spine density and a reduction of immature spines. After the treatment with GLP-1 (9-36), the long-term potentiation failure and cognitive impairments are rescued in Ts65Dn. The results suggest that GLP-1 (9-36) treatment may have significant therapeutic benefits for DS where neuronal oxidative distress is pronounced.

Thus, investigating the link between GLP-1 signaling, mitochondrial health, and neurodegenerative conditions such as AD and DS could provide valuable insights into novel treatments that target the underlying metabolic dysfunctions in these diseases.

## Incretin Mimetics for Alzheimer’s Disease: Bridging Metabolic and Cognitive Health

Current treatments for AD provide only limited symptom relief, which has prompted interest in evaluating in the preclinical and clinical phases existing drugs, particularly the incretin mimetics used in the treatment of T2DM. It is therefore not surprising that these findings have sparked interest in exploring GLP-1 analogs as a potential treatment for AD (Nowell et al., 2023b).

GLP-1RAs, also known as incretin mimetics, are released in response to glucose and enhance insulin secretion by pancreatic β-cells while suppressing glucagon release, thereby lowering postprandial blood sugar levels. Beyond their role in glycemic regulation, GLP-1RAs exhibit neurotrophic and neuroprotective properties, such as improving long-term potentiation and promoting synaptic growth (Ferrari et al., 2022; Nowell et al., 2023a). Several lines of evidence suggest that these agents can restore cognitive function, reduce amyloid plaque deposition, prevent synaptic loss, and mitigate neuronal inflammation. They also protect hippocampal neurons from Aβ_1–42_-induced cell death, decrease APP and Aβ levels, and counteract tau hyperphosphorylation by modulating GSK-3β activity (An et al., 2015). As explained in the above section, native incretin hormones such as GLP-1 and gastric inhibitory polypeptide (GIP) have short half-lives due to rapid degradation by DPPIV, long-acting GLP-1RAs and DPPIV inhibitors have been developed (Ferrari et al., 2022). **Tables 1** and **2** provide a summary of the studies on GLP-1RAs discussed below.

**Table 1:** Results of *in vitro* and *in vivo* preclinical investigations in AD and insulin-resistant animal models

Drug	*In vitro/in vivo* model	Dosage	Effect	Reference
Exenatide	Aβ challenged rats	5 μg/kg	Reduced memory impairment, decreased Aβ aggregation in the hippocampus and frontal cortex, and lowered mitochondrial toxicity in the brain	Garabadu and Verma, 2019
	5xFAD mice	100 μg/kg	Improved spatial memory and learning ability, reduced hippocampal Aβ1–42 deposits, and reduced oxidative distress	An et al., 2019
	3xTg-AD high-fat diet-fed mice	500 μg/kg	Reversed negative changes in brain-derived neurotrophic factor signaling and neuroinflammation. No alterations in systemic metabolism nor in cognitive performance	Bomba et al., 2019
Liraglutide	hTauP301L mice	500 μg/kg	Improved neurological function and lowered phospho-tau pathology	Hansen et al., 2016
	α-Pyrrolidinononanophenone-administered rats	47 and 94 μg/kg	Counteracted α-Pyrrolidinononanophenone-induced deficits in environmental learning and memory by ameliorating mitochondrial function	Noruzi et al., 2024
Semaglutide	SH-SY5Y cells exposed to Aβ25–35	10 nM	Pro-autophagic effects and demonstrated anti-apoptotic effect by inhibiting the Aβ25–35-induced Bax pathway	Chang et al., 2020
	3xTg-AD mice	0.1 mg/kg	Ameliorated cognition and glucose metabolism dysfunction	Wang et al., 2023
Tirzepatide	GLP-1R knockout mice	10 nmol/kg	Improved insulin sensitivity by enhancing glucose disposal in white adipose tissue	Samms et al., 2021
LY3437943	Obese mice	10 nmol/kg	Body weight loss and increased energy expenditure through glucagon receptor activation	Coskun et al., 2022

Atg5: Autophagy related 5; Aβ: amyloid-β; Mfn2: Mitofusin 2; mito-LC3II: mitochondrial microtubule-associated proteins 1A/1B light chain 3B II; Smac: second mitochondria-derived activator of caspase; x-IAP: X-linked inhibitor of apoptosis protein.

**Table 2:** Ongoing evidence from the latest clinical trials in patients

Drug name	Number of patients	Dosage	Outcome	Reference
Exenatide	57 with AD or MCI	Twice-daily injection pen from 5 to 10 mg over 18 mon	Reduced neuronal EV Aβ42 but no improvement in cognitive outcomes	Mullins et al., 2019
	32 with MCI	Once-weekly subcutaneous injection of 2 mg over 32 wk	No beneficial effect on cognitive performance	Dei Cas et al., 2024
	194 with PD	Once-weekly of 2 mg over 96 wk	No difference from placebo, suggesting that there is no slowing of PD progression	Vijiaratnam et al., 2021
Liraglutide	204 with AD	Once-daily subcutaneous injection of 1.8 mg over 12 mon	Improved cognitive function and MRI volume in AD subjects	Femminella et al., 2019
Semaglutide	1800 with AD or MCI	Once-daily oral administration from 3 to 14 mg over 12 mon	The evoke and evoke+ read-outs are expected in 2026	NCT04777396; NCT04777409
Tirzepatide	2539 with a BMI of 30	Once-weekly subcutaneous injection from 5 to 15 mg over 72 wk	Substantial and sustained reductions in body weight	Jastreboff et al., 2022
LY3437943	72 with T2DM	Once-weekly subcutaneous injection from 0.5 to 12 mg over 12 wk	Significant reduction in plasma glucose and HbA1c levels in the higher-dose groups, and a dose-dependent decrease in body weight	Urva et al., 2022

AD: Alzheimer’s disease; Aβ: amyloid-β; BMI: body mass index; EV: extracellular vesicle; MCI: mild cognitive impairment; MRI: magnetic resonance imaging; PD: Parkinson’s disease; T2DM: type 2 diabetes mellitus.

Exenatide is a synthetic form of a naturally occurring GLP-1RA found in the saliva of the Gila monster; it was approved by the Food and Drug Administration in April 2005 as the first drug in the class of incretin mimetics, and as adjunctive therapy to metformin or a sulfonylurea for the improvement of glycemic control in patients with T2DM who have not achieved adequate glycemic control (Segal et al., 2007). Exenatide, a GLP-1 mimetic, helps in regulating blood glucose levels by mimicking the action of endogenous GLP-1. It is resistant to degradation by DPPIV and binds to GLP-1Rs, although its half-life in the bloodstream is relatively short, lasting approximately 15 hours following subcutaneous injection. By activating GLP-1Rs, exenatide stimulates insulin secretion in response to food intake, suppresses glucagon release, and delays gastric emptying. These combined effects contribute to reducing blood glucose levels and improving overall glycemic control (Nadkarni et al., 2014).

In support of the the hypothesis that exenatide may alleviate AD symptoms and promoting insulin-mediated neuroprotective effects, Garabadu and Verma (Garabadu and Verma, 2019) demonstrated that exendin-4 reduces mitochondrial toxicity in a Aβ-induced AD rodents, with this effect mediated through the PI3K/ AKT signaling pathway. This finding was further supported by a study on 5xFAD mice conducted in 2020 by An et al. (2019), which showed that exendin-4 treatment mitigated cognitive dysfunction, prevented Aβ deposits, and maintained mitochondrial integrity.

Moreover, in 2019, Bomba et al. evaluated the effects of exenatide treatment over three months in 3xTg-AD mice subjected to an HFD for six months, with the final aim of assessing whether changes in systemic metabolism, were associated with an improvement in brain insulin signaling, AD-related neuropathology, as well as in cognitive performance. Collected results showed that exenatide reverses the negative changes in brain-derived neurotrophic factor signaling. In 3xTg-AD mice on an HFD, neuroinflammatory status remained unchanged, with no impact on systemic metabolism or cognitive function.

While preclinical studies suggest GLP-1RAs could support cognitive function by reducing inflammation and promoting neurogenesis, clinical trials in AD have yielded mixed results. Some trials with exenatide have failed to show significant cognitive improvement in AD (Mullins et al., 2019), particularly in patients with MCI (Dei Cas et al., 2024) or Parkinson’s disease (Vijiaratnam et al., 2021). This latter did not show a benefit over placebo, suggesting the drug does not slow disease progression. The disappointing outcome contrasts with earlier promising results, underscoring the complexity of treatment of Parkinson’s disease and the need for further investigation into diabetes-related drugs (as well as for the complexity of AD). This is the reason why other GLP-1 receptor agonists remain under investigation.

Liraglutide is an example of a recombinant GLP-1 analog with delayed absorption and a longer plasma half-life of over 13 hours due to its binding to albumin. It has fewer side effects and better effectiveness in reducing glycated hemoglobin and fasting blood glucose than short-acting GLP-1R agonists (Ferrari et al., 2022). In AD, liraglutide has been shown to enhance brain glucose metabolism and functional connectivity in small-scale pilot studies. In 2016, Hansen et al. conducted a study of liraglutide in a mouse model of tauopathy in which they showed that the drug acts on GLP-1 by reducing pathology-specific tau phosphorylation and improving motor function, as well as neurological function. The potential of liraglutide as an AD treatment was investigated in a phase IIb, double-blind, randomized, placebo-controlled trial conducted at multiple centers in the United Kingdom. In this study, 204 adults with mild to moderate AD received daily subcutaneous injections of liraglutide or a placebo for 12 months. No differences in brain glucose metabolism were observed between the groups, although participants treated with liraglutide showed improvement in cognitive function (Femminella et al., 2019). Moreover, a study conducted by Noruzi et al. in 2024 demonstrated that administering liraglutide for 28 days efficiently alleviated spatial learning and memory impairments in rats, as well as brain mitochondrial dysfunction induced by α-pyrrolidinononanophenone.

As the authors shown, an increase of α-pyrrolidinononanophenone damages mitochondrial biogenesis by triggering cytochrome c, and causing mitochondrial swelling in the brain, leading to an altered adenosine diphosphate/ATP ratio in rats. Liraglutide treatment was found to mitigate the damage to the mitochondrial outer membrane and reduce cytochrome c release in α-pyrrolidinononanophenone-treated rats.

Semaglutide is a modified version of human GLP-1, sharing 94% of its structure, and these modifications enhance its resistance to breakdown by DPP-IV enzyme and improve its binding to albumin. While human GLP-1 has a very short half-life of just one to two minutes due to rapid DPP-IV inactivation, semaglutide has a much longer half-life of 165 to 184 hours, allowing for weekly dosing (Jung and Jung, 2022). This drug showed pro-autophagic effects by increasing the expression of LC3II, Atg7, Beclin-1, and p62 and demonstrated an anti-apoptotic effect by inhibiting the Aβ_25–35_-induced Bax pathway in an *in vitro* model of AD, i.e., SH-SY5Y cells exposed to Aβ_25–35_ (Chang et al., 2020). Moreover, Wang et al. (2023) demonstrated that semaglutide ameliorates cognition and glucose metabolism dysfunction in the 3xTg mouse model of AD. Two large phase III clinical trials are currently underway: evoke and evoke+ (NCT04777396 and NCT04777409, respectively), conducted by Novo Nordisk company with completion scheduled between 2025 and 2026. Each trial includes 1840 participants with MCI or mild dementia from AD, who will be randomly assigned to receive daily oral semaglutide or a daily oral placebo for 156 weeks.

More recently, dual and triple agonists targeting both GLP-1R and GIP receptor have been developed to increase benefits while reducing side effects. An example of a GLP-1R/GIP receptor dual agonist is tirzepatide.

Tirzepatide is a synthetic peptide composed of 39 amino acids, with a structure derived from native GIP. The inclusion of a 20-carbon fatty diacid moiety in tirzepatide extends its half-life to 5 days, enabling once-weekly injections. GIP and GLP-1 co-administration has demonstrated synergistic effects in reducing body weight, fat mass, and food intake in animal models of obesity and diabetes. Samms et al. (2021) demonstrated that tirzepatide acts better than GLP-1RA as an insulin sensitizer in obese mice. The authors compared the effects of tirzepatide in obese wild-type and GLP-1R knockout mice, observing that, in the absence of GLP-1R-mediated weight loss, tirzepatide still enhanced insulin sensitivity by promoting glucose uptake in white adipose tissue. Tirzepatide is a new drug approved by the Food and Drug Administration for the treatment of T2DM that leads to significant improvement in glycemic control and weight reduction in patients (Jastreboff et al., 2022), maximizing benefits similar to GLP-1 drugs such as semaglutide (France and Syed, 2024). In general, while both semaglutide and tirzepatide are effective for managing T2DM and supporting weight loss, dual action of tirzepatide on GLP-1 and GIP receptors provides a potentially broader range of benefits compared to semaglutide’s single receptor activation.

LY3437943 is a promising triple agonist currently in clinical trials. Its unique mechanism of action aims to provide comprehensive benefits for patients with T2DM and obesity by targeting three key metabolic pathways: GLP-1, GIP, and glucagon. This triple activation aims to enhance glycemic control, promote weight loss, and improve lipid metabolism, potentially offering comprehensive treatment for metabolic disorders. In 2022, Coskun et al. have demonstrated that LY3437943 can reduce body weight through increased energy expenditure and reduced calorie intake in obese mice, whether Urva et al. (2022) have conducted a 12-week study and have examined the safety, pharmacokinetics, and pharmacodynamics of multiple weekly doses of LY3437943 in individuals with T2DM. This early-phase study, funded by Eli Lilly and Company, demonstrated an acceptable safety profile for LY3437943 with its pharmacokinetic properties. In light of these results, because of significant reductions in both glucose levels and body weight, the study supports its progression to phase 2 development. Furthermore, the success of LY3437943 in clinical development could pave the way for new therapeutic options in the management of these complex metabolic conditions. This trial is registered at ClinicalTrials.gov, NCT04143802 (Urva et al., 2022).

As mentioned above, brain insulin signaling plays a key role in memory and is considered a promising target for preventing and treating memory impairments, including AD. For this reason, intranasal insulin administration is thought to enhance synaptic plasticity, boost regional glucose uptake, and reduce AD-related neuropathology, improving memory and cognitive performance (Hallschmid, 2021). However, although it has been shown to be effective and safe, recent large-scale clinical trials highlight the need for further research to better assess the potential of insulin in alleviating AD. These include exploring sex differences in treatment response and improving delivery methods to maximize its potential in AD therapy. **Table 3** summarizes some of the most compelling clinical trials conducted in the past decade.

**Table 3:** Clinical trials of intranasal insulin administration

Number of patients	Dosage	Outcome	Reference
36 with MCI or mild to moderate AD	Daily 40 IU for 4 mon	Better memory after two months, preserved volume on MRI, reduced tau-P181/Aβ42 ratio	Craft et al., 2017
289 with MCI or AD	Daily 40 IU for 12 mon	Intranasal insulin treatment did not result in any cognitive or functional improvements	Craft et al., 2020
49 with MCI or AD	Twice-daily 20 IU for 12 mon	Improved inflammatory markers in cerebrospinal fluid, better cognitive performance, slower symptom progression, and improvements in amyloid and tau ratios in cerebrospinal fluid	Kellar et al., 2022
80 with metabolic syndrome or MCI	Twice-daily 20 IU for 12 mon (combined with semaglutide)	Improved cerebral perfusion and insulin signaling in the brain, two factors associated with cognitive decline, which can potentially improve cognitive function	Davidy et al., 2024

AD: Alzheimer’s disease; Aβ: amyloid-β; MCI: mild cognitive impairment; MRI: magnetic resonance imaging.

GLP-1RAs have demonstrated a range of beneficial effects on mitochondria. By promoting insulin action at the molecular level, these drugs increase the efficiency of ATP production, the main energy source of the cell, contributing to improved cellular performance, enhancing mitochondrial respiration, and improving neuronal function (Carlessi et al., 2017; Galizzi and Di Carlo, 2022). Additionally, these drugs help in preventing the production of ROS (Rowlands et al., 2018). Thus, the reduction of oxidative distress, in turn, prevents cellular damage and improves overall cellular health, helping to avoid mitochondrial damage—a key factor in neurodegenerative progression (Lanzillotta et al., 2019; Barone et al., 2021; Galizzi and Di Carlo, 2022). Moreover, oxidative distress can impair insulin signaling pathways, leading to insulin resistance by interfering with the activation of PI3K and AKT, both crucial for glucose uptake. By decreasing oxidative distress, GLP-1RAs support more effectively insulin signaling (Oh and Jun, 2017). These drugs also play a vital role in promoting mitochondrial biogenesis, essential for cellular health. This effect is mediated by various signaling pathways, including the activation of PGC-1α, a key regulator of mitochondrial biogenesis. This process is crucial for maintaining a healthy and functional mitochondrial population (Galizzi and Di Carlo, 2022; Wu et al., 2022). Consequently, the formation of new mitochondria is vital for preserving cellular health and counteracting neuronal function loss in AD. Another significant action of GLP-1RAs is their ability to protect cells from apoptosis induced by mitochondrial dysfunction. This protective effect is particularly relevant in pancreatic beta cells, where preventing apoptosis helps maintain cell mass and insulin functionality (Chang et al., 2020; Galizzi and Di Carlo, 2022), as well as in AD, where reducing brain cell loss could slow the cognitive decline associated with the disease. It is reasonable that GLP-1RAs represent a promising class of drugs with beneficial effects on mitochondria, potentially aiding in the management of neurodegenerative conditions such as AD and DS (Day et al., 2019). By improving mitochondrial function, reducing oxidative distress, promoting mitochondrial biogenesis, preventing apoptosis, and enhancing tissue sensitivity to insulin, these drugs offer potential therapeutic benefits for neuronal health and slowing the progression of these diseases.

Despite the very promising preclinical data, clinical research is still in its infancy. Future studies will be crucial to determine the long-term efficacy and safety of these drugs as neuroprotective treatments. Currently, there is no definitive cure for AD, but there are medications and therapies available that can help manage symptoms and temporarily slow the progression of the disease. Research on AD is very active, with many drugs undergoing clinical trials, and efforts continue in hopes of developing more effective treatments or possibly a cure, as well as improving the quality of life for individuals with DS (Day et al., 2019). Further investigation through large clinical trials is necessary to assess the use of GLP-1, GIP, and dual/triple RAs, along with intranasal insulin. These compounds could potentially offer disease-modifying treatments for various neurodegenerative disorders. Targeting incretin and insulin receptors in the brain seems promising for these conditions, possibly due to neuroprotective effects such as reducing cell death and inflammation, while promoting neurogenesis.

In conclusion, ongoing research will continue to explore and clarify the role of GLP-1RAs in these and other conditions. Current studies on new GLP-1RAs and innovative formulations promise to further expand therapeutic possibilities and improve patients’ overall health and well-being.

## Future Perspectives

The data presented in this review underscore the intricate interplay between insulin resistance, mitochondrial dysfunction, and neurodegeneration, highlighting their critical roles in the pathogenesis of AD and DS. Impaired insulin signaling in the brain not only disrupts energy metabolism and synaptic plasticity but also contributes to oxidative distress and chronic inflammation, exacerbating neuronal damage. Simultaneously, mitochondrial dysfunction—characterized by defective biogenesis, impaired dynamics, and reduced clearance of damaged organelles—worsens metabolic stress and promotes the deposits of neurotoxic proteins such as Aβ and hyperphosphorylated Tau. Future research should prioritize the development of integrated therapeutic strategies targeting both insulin resistance and mitochondrial dysfunction. GLP-1RAs, originally designed for diabetes treatment, are emerging as promising neuroprotective agents due to their ability to enhance insulin signal transduction, reduce oxidative distress, and mitigate neuroinflammation. In parallel, intranasal insulin administration represents a compelling approach to bypass peripheral insulin resistance and directly enhance brain metabolism, potentially benefiting both AD and DS populations. However, despite encouraging preclinical and early clinical findings, further investigations are required to evaluate the long-term efficacy and safety of these interventions. In particular, studies should explore their impact on genetic models of AD, such as DS, where neurodegeneration manifests early and is particularly severe. A deeper characterization of biomarkers linked to mitochondrial dysfunction and insulin resistance will be essential to facilitate the development of personalized therapeutic approaches. The integration of machine learning approaches for biomarker discovery and patient stratification could further enhance the precision of these interventions. Ultimately, the convergence of metabolic and neurodegenerative research fields holds great potential for advancing therapeutic innovation. By refining our understanding of the molecular pathways governing insulin signaling and mitochondrial function, we may pave the way for novel disease-modifying strategies capable of mitigating neurodegeneration and improving cognitive health in at-risk populations.

## Data Availability

*Not applicable*.
